# 196. Activity Impairment and Health-Related Quality of Life Associated with an Uncomplicated Urinary Tract Infection Among US Females

**DOI:** 10.1093/ofid/ofab466.196

**Published:** 2021-12-04

**Authors:** Jeffrey Thompson, Alen Marijam, Fanny S Mitrani-Gold, Jonathon Wright, Ashish V Joshi

**Affiliations:** 1 Kantar Health, New York, NY, USA, New York, New York; 2 GlaxoSmithKline plc., Collegeville, PA, USA, Collegeville, Pennsylvania; 3 GlaxoSmithKline plc, Collegeville, PA, USA, Chicago, Illinois

## Abstract

**Background:**

Uncomplicated urinary tract infections (uUTI) are among the most common infections in women; however, there are few data on the impact of uUTIs on daily activity and health-related quality of life (HRQoL).

**Methods:**

This was a prospective, cross-sectional survey of US females aged ≥ 18 years with a self-reported uUTI in the 60 days prior to participation. Participants were included if they received oral antibiotic treatment and participated in surveys fielded by Dynata, Lucid/Federated, or Kantar Profiles. See **Table 1** for inclusion/exclusion criteria. Study objectives were to describe activity impairment (using the Activity Impairment Assessment [AIA]) and HRQoL (assessed with Short Form 36 version 2, Physical Component Score [PCS], Mental Component Score [MCS], and health utility index [SF-6D]) associated with uUTI. After screening, participants completed an online questionnaire on their most recent uUTI. Outcomes were reported with descriptive statistics, chi-squared tests, and t-tests. Analysis of HRQoL used 1:1 propensity score matching to compare to a matched US population from the 2020 National Health and Wellness Survey.

Table 1. Inclusion and exclusion criteria

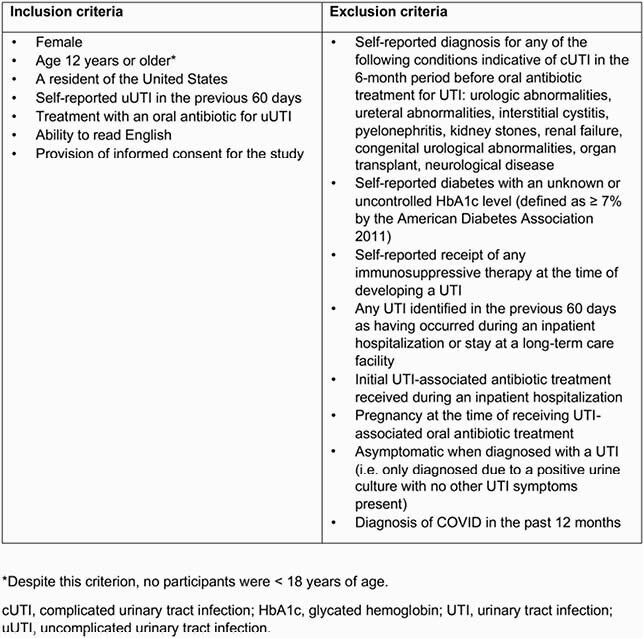

**Results:**

In total, 375 participants completed the questionnaire. Common impaired activities were: sexual intercourse (66.9%), sleep (60.8%), exercise (52.3%), housework (51.5%), and social activities (46.9%; **Table 2**). Overall mean AIA score was 11.1/20 (higher score = more impairment). Most participants (58.7%) had a PCS that was the same or better than the matched population, while for MCS, most participants (52.8%) had scores well below the matched population average. Overall PCS, MCS, and SF-6D composite scores were 46.5, 40.0, and 0.63, respectively; these outcomes were significantly worse compared to the matched population, most notably MCS (**Table 3**). Stratification by number of antibiotics used revealed statistically significant differences in the effect of uUTI on exercise, PCS, SF-6D (based on use of 1 or ≥ 3 therapies), and on sleep (based on use of 2 or ≥ 3 therapies; **Table 4**).

Table 2. Activities impacted by uUTI

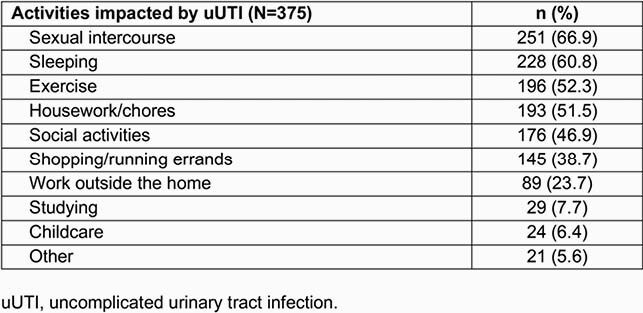

Table 3. Matched analysis of SF-36v2-measured HRQoL outcomes

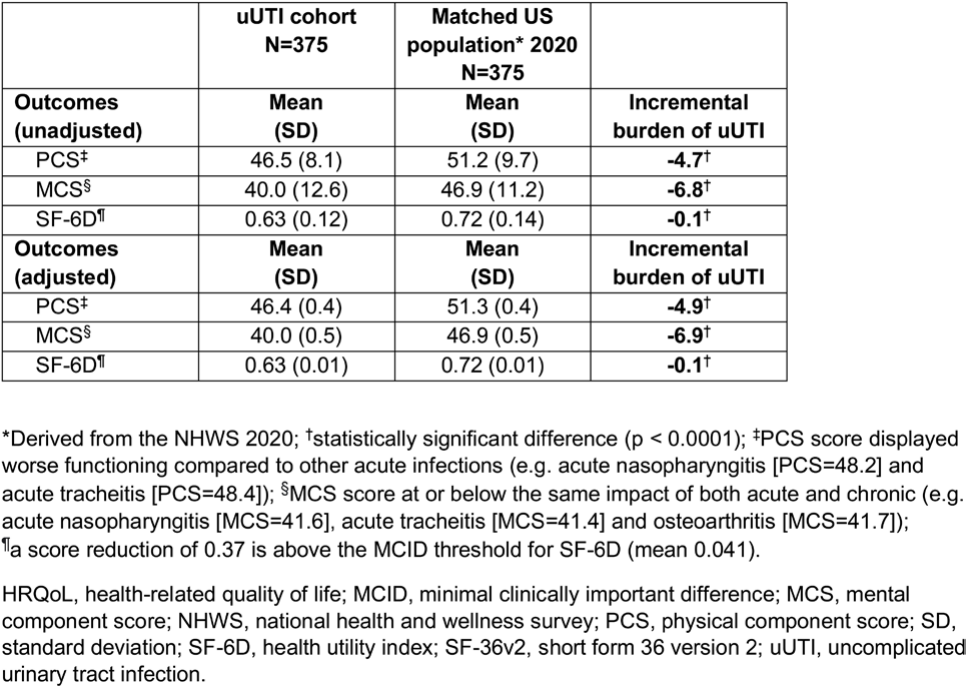

Table 4. Outcomes stratified by number of oral antibiotics used to treat last uUTI

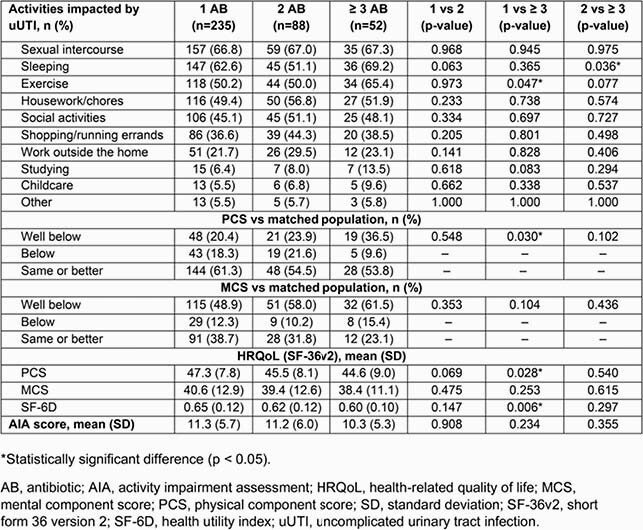

**Conclusion:**

uUTIs are significantly associated with adverse patient outcomes for daily activities and HRQoL, compounded by suboptimal treatment evident by the use of multiple antibiotics. MCS was notably affected, which is important as this is not often studied in uUTI.

**Disclosures:**

**Jeffrey Thompson, PhD**, **Kantar Health** (Employee, Employee of Kantar Health, which received funding from GlaxoSmithKline plc. to conduct this study) **Alen Marijam, MSc**, **GlaxoSmithKline plc.** (Employee, Shareholder) **Fanny S. Mitrani-Gold, MPH**, **GlaxoSmithKline plc.** (Employee, Shareholder) **Jonathon Wright, BSc**, **Kantar Health** (Employee, Employee of Kantar Health, which received funding from GlaxoSmithKline plc. to conduct this study) **Ashish V. Joshi, PhD**, **GlaxoSmithKline plc.** (Employee, Shareholder)

